# Virological Effectiveness of Dolutegravir Plus Darunavir in People with Multi-Drug-Resistant HIV: Data from the PRESTIGIO Registry

**DOI:** 10.3390/v17091158

**Published:** 2025-08-24

**Authors:** Filippo Lagi, Michele Bellomo, Riccardo Lolatto, Filippo Ducci, Seble Tekle Kiros, Vincenzo Spagnuolo, Rebecka Papaioannu Borjesson, Tommaso Clemente, Leonardo Calza, Marcello Feasi, Emanuele Focà, Andrea Giacomelli, Roberto Gulminetti, Barbara Menzaghi, Antonella Castagna, on behalf of the PRESTIGIO Study Group

**Affiliations:** 1 Infectious and Tropical Diseases Unit, Careggi University Hospital, 50134 Florence, Italy; 2 Infectious Diseases, IRCCS San Raffaele Scientific Institute, 20127 Milan, Italy; 3 Department of Experimental and Clinical Medicine, University of Florence, 50134 Florence, Italy; 4 IRCCS San Raffaele Hospital, Vita-Salute San Raffaele University, 20127 Milan, Italy; 5 Unit of Infectious Diseases, Department of Medical and Surgical Sciences, S. Orsola Hospital, “Alma Mater Studiorum” University of Bologna, 40126 Bologna, Italy; 6 Department of Infectious Diseases, E.O. Ospedali Galliera, 16128 Genoa, Italy; 7 Unit of Infectious and Tropical Diseases, Department of Clinical and Experimental Sciences, University of Brescia and ASST Spedali Civili Hospital, 25123 Brescia, Italy; 8 Department of Biomedical and Clinical Sciences, Università Degli Studi di Milano, 20122 Milan, Italy; 9 III Infectious Diseases Unit, ASST Fatebenefratelli Sacco, 20157 Milan, Italy; 10 Fondazione IRCCS Policlinico San Matteo, University of Pavia, 27100 Pavia, Italy; 11 Unit of Infectious Diseases, ASST della Valle Olona, 21052 Busto Arsizio, Italy

**Keywords:** dolutegravir, darunavir, multi-drug-resistant HIV, PRESTIGIO

## Abstract

Background: Data on the use of dolutegravir (DTG) plus boosted darunavir (DRV/b) in people with 4-class drug-resistant HIV (4DR-PWH) are limited. This study assessed the virological effectiveness of DTG + DRV/b in this population using real-world data from the PRESTIGIO Registry. Methods: We compared three regimen groups: dual DTG + DRV/b (DODA), DTG + DRV/b plus an additional antiretroviral drug (DODA + Other), and regimens excluding DTG + DRV/b (NO-DODA). Virological failure (VF) was defined as ≥2 HIV-RNA values ≥ 50 copies/mL or 1 ≥ 1000 copies/mL. Mixed-effects logistic regression was used to assess VF, adjusting for antiretroviral therapy (ART) duration, age, number of fully active drugs, sex at birth, and nadir CD4+. Individuals could switch regimens during follow-up. Results: Among 249 4DR-PWH (median follow-up: 8.7 years), 844 ART regimens were analyzed: 72 (8.5%) DODA, 264 (31.3%) DODA + Other, and 508 (60.2%) NO-DODA. Compared to NO-DODA, the odds of VF were 77% and 35.9% lower with DODA and DODA + Other, respectively. Notably, in the DODA group, DTG and DRV/b were fully active in only 63.9% and 47.2% of the cases, respectively. Conclusions: DTG + DRV/b regimens were associated with a significantly lower risk of virological failure, even when drug activity was partial. This strategy remains a valuable option for managing multi-drug-resistant HIV.

## 1. Introduction

In recent years, many new therapeutic options for people with HIV (PWH) have led to high rates of virological suppression and improved life expectancy, almost closing the gap in the general population [1]. Globally, approximately 76% of PWH on antiretroviral therapy (ART) achieve virological suppression. However, substantial regional disparities remain, with lower rates reported in some low- and middle-income countries [2]. According to the last available data in Italy, it is estimated that 93% of PWH achieved virological suppression [3]. However, concerns remain about some populations, such as heavily treatment-experienced (HTE) individuals, who have a long history of previous ART exposure and fewer available therapeutic options [4]; therefore, they may require more complex antiviral regimens to achieve virological suppression. This increases the risk of toxicities, potential drug–drug interactions (DDIs) poor adherence, and consequently virological failure (VF) [5,6,7,8]. However, data on this population are scarce. Some insights into HTE individuals with documented resistance to all major antiretroviral classes come from studies such as the BRIGHTE (fostemsavir) [9], CAPELLA (lenacapavir) [10], and TMB-301 (ibalizumab) [11]. However, the number of participants with profound multi-drug resistance remains limited in all these studies. 

The PRESTIGIO Registry is the Italian multicenter Registry of PWH with documented 4-class drug resistance (4DR), established in 2017 to collect observational clinical and virological data on this population [12].

Dolutegravir (DTG) and boosted darunavir (DRV/b) have been used in HTE individuals given their high efficacy and high genetic barrier. Several studies have evaluated dual therapy with DTG + DRV/b in this population [13,14,15,16,17,18,19], although the majority of these studies included only individuals with documented full activity of DRV/b and integrase strand transfer inhibitors (INSTIs). Data on the DTG + DRV/b combination in the 4DR population are scarce. Therefore, our study aimed to evaluate the virological efficacy of DTG plus DRV/b in individuals with 4DR-PWH in a real-world setting.

## 2. Materials and Methods

The primary objective of the study was to evaluate the effectiveness of the combination of DTG and DRV/b in terms of virological failure among 4DR-PWH. Given the high frequency of regimen switches in this population, our primary analytical approach focused on regimens rather than individuals. In addition, we decided not to use a traditional ‘time-to-event’ approach, as this would have ignored important information collected after virological failure. By analyzing the full course of each regimen, including what happened after a failure, we aimed to better reflect the complex treatment history of these individuals.

Specifically, we categorized ART regimens into three groups: (i) dual therapy with DTG plus DRV/b (DODA); (ii) DTG plus DRV/b plus ≥1 additional ARV drug (DODA + Other); and (iii) regimens not containing both DTG and DRV/b (NO-DODA). This regimen-based approach allows us to better capture the clinical impact of using the DTG + DRV/b combination across different treatment strategies.

In the PRESTIGIO Registry, individuals were followed from the date of first evidence of 4DR (baseline) until death, loss to follow-up, or 30 May 2024 (freezing date). However, follow-up time was also calculated separately for each ART regimen of interest for this analysis. Specifically, follow-up for a given regimen (DODA, DODA + Other, or NO-DODA) began at the start of that regimen and ended at its discontinuation. Therefore, the same person could contribute to more than one regimen group over time and switch between groups multiple times during the overall follow-up period.

### 2.1. Definitions and Statistical Analysis

Virological failure (VF) was defined as ≥2 consecutive HIV-RNA measurements ≥ 50 copies/mL or a single measurement ≥ 1000 copies/mL. Based on this definition, each regimen was classified at each HIV-RNA measurement as either 0 (no virological failure) or 1 (virological failure). Any subsequent observation showing HIV-RNA > 50 copies/mL following an initial VF is also classified as a VF (coded as 1). All centers consistently adopted a lower limit of detection of 50 copies/mL during the study period.

The Genotypic Susceptibility Score (GSS) was calculated for each drug at the start of each regimen included in the analysis, with a value of 0 indicating full drug resistance and 1 indicating full drug activity, based on cumulative genotypic resistance testing (GRT) interpreted according to the Stanford HIV database algorithm (version 9.6, hivdb.stanford.edu accessed on 1 September 2024). If a new genotypic resistance test was performed during follow-up, its results were incorporated into the cumulative GRT and used to calculate the GSS for subsequent regimens.

In cases where the INSTI GRT was unavailable, the GSS for DTG was determined as follows: for individuals who experienced virological failure with raltegravir (RAL) or elvitegravir (EVG), full resistance (GSS value 0) was assumed for both RAL and EVG, while full activity (GSS value 1) was assumed for DTG and bictegravir (BIC); for those who failed on BIC or DTG, full resistance was assigned to RAL, EVG, and to the failed second-generation INSTI (BIC or DTG), while susceptibility was assumed for the other drug.

Characteristics were described as median (interquartile range) or frequency (percentage); comparisons were made using Fisher’s exact test or Mann–Whitney U test, as appropriate.

The relationship between DTG + DRV/b and VF was analyzed using a mixed-effects logistic regression model. We adopted an event-based approach in which each virological failure was evaluated in relation to the regimen in place at the time of failure, adjusting the regression for the following prespecified variables: ART duration, number of fully active ARVs, sex at birth, age and nadir CD4+. Individual failure predisposition was estimated with a random intercept [20].

### 2.2. Ethics

This study involved only human participants. The PRESTIGIO Registry was approved by the Ethics Committee of the coordinating center (IRCCS San Raffaele Scientific Institute, Milan, Italy; protocol number 41/int/December 2017) and by the ethics committees of all participating centers.

## 3. Results

We present the results of this study by first describing individual-level data to characterize the cohort, followed by the main analysis focused on treatment regimens, which represents the primary objective of the study.

### 3.1. 4DR-PWH Included in the Analysis

At the time of the freezing date, we included in the analysis all individuals enrolled in the PRESTIGIO Registry, corresponding to the entire cohort of 249 4DR-PWH with a median follow-up of 8.7 years (5.9–11.5) per person and a total follow-up time of 2147.9 years. In 47 4DR-PWH (18.9%), interruption of follow-up was observed for the following reasons: 33 due to death, 8 due to transfer to another center, and 6 due to unknown causes. During follow-up, 60 individuals (24.1%) received DODA, 136 (56.4%) were treated with DODA + Other combinations, and 181 (72.7%) were exposed to NO-DODA. These figures refer to individuals who have been exposed to each regimen at least once. As patients could switch regimens, the total number of regimens analyzed is higher and includes multiple entries per person (see below). At baseline, the 4DR-PWHs in virological suppression (viremia < 50 copies/mL) were 23 (9.2%). No 4DR-PWH failed DTG and subsequently switched to BIC or vice versa.

A total of 4377 HIV-RNA measurements were recorded in the registry. During follow-up, each participant had a median of 15 viral load measurements (IQR, 10–21), with a median interval of 133 days (IQR, 75–202) between consecutive measurements. The resistance tests used to calculate the GSS totaled 1036. The median number of those tests per patient was 3 (range 1–6). Among the 249 PWH included in the study, 62 had no documented INSTI resistance but experienced virological failure while on an INSTI-based regimen: 49 with raltegravir, 4 with elvitegravir, and 9 with dolutegravir.

### 3.2. ART Regimens Included in the Analysis

Among the 249 4DR-PWH described above, a total of 844 antiretroviral treatment regimens were analyzed. These were categorized into three groups: 72 (8.5%) DODA, 264 (31.3%) DODA + Other, and 508 (60.2%) NO-DODA. Among the 508 regimens classified as No-DODA, 207 (40.7%) contained DRV/b and 179 (35.2%) included DTG (Table 1). Overall, the DTG + DRV/b combination, either as dual therapy or in combination with at least one additional antiretroviral agent, was used in 336 out of 844 treatment regimens, accounting for 39.8% of all cases. The corresponding median duration of the three regimens (in years) was 4.2 (IQR 1.7–5.7), 1.8 (0.6–3.5), and 1.3 (0.5–2.8), respectively (Table 1). Although prescribed less frequently, DODA showed significantly longer durability than DODA + Other and NO-DODA (*p* < 0.001 for both comparisons).

Table 1 compares various treatment regimens in terms of duration, cumulative exposure, number of individuals exposed, and individual/combined activity of the included drugs.

We observed significant differences in the number of fully active drugs included at the initiation of the regimen. In the DODA group, in 50.0% of the cases in which it was used, one of the two drugs (DRV or DTG) was fully active, while both were active in 30.6% of cases. In the DODA + Other group, more than two fully active drugs were included in 11.0%. Conversely, in 39.6% of the cases where NO-DODA was used, no fully active drugs were present.

When specifically analyzing the individual activity of DTG and DRV/b at the time of their inclusion in the regimen, we found that both drugs were fully active (GSS = 1) significantly more often in the DODA group compared to the others. DTG was fully active in 63.9% of DODA cases, versus 46.2% in DODA + Other (*p* = 0.001) and 49.7% in NO-DODA (*p* < 0.001). Similarly, DRV/b showed full activity in 47.2% of DODA uses, compared to 26.5% in DODA + Other (*p* = 0.011) and 23.7% in NO-DODA (*p* = 0.050).

**Figure 1:**
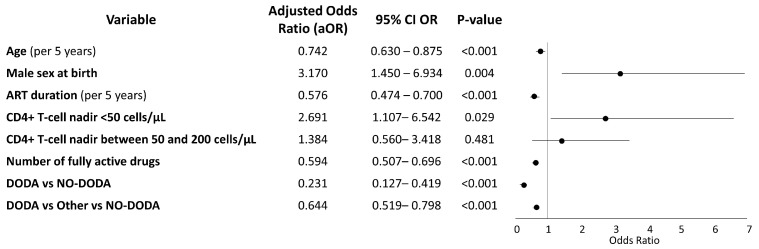
Multivariable logistic regression analysis of factors associated with virological failure. DODA: regimens including DRV/b + DTG only. DODA + Other: regimens including DTG and DRV/b plus ≥1 additional antiretroviral drug. NO-DODA: regimens not including the combination of DTG and DRV/b.

In the adjusted logistic regression analysis, both DODA and DODA + Other regimens were associated with significantly lower odds of virological failure compared to the NO-DODA regimen. Specifically, DODA was associated with a 77% reduction in the odds of failure (aOR 0.23; 95% CI: 0.13–0.42; *p* < 0.001), while DODA + Other was associated with a 36% reduction (aOR 0.64; 95% CI: 0.52–0.80; *p* < 0.001). Each fully active ARV in the regimen decreased the odds of VF by 40% (aOR 0.594; 95%CI 0.507–0.696; *p* < 0.001). Older age and longer ART duration reduced the odds of VF (Figure 1). A CD4 nadir < 50 cells/mm^3^ was associated with higher odds of VF (Figure 1).

In the DODA group, the odds of virological failure were lower than those in the other groups, despite DTG being fully active in 63.9% of cases, DRV/b in 47.2%, and full activity for both drugs in 30.6% of cases (Table 1). Even in the DODA + Other group, the odds of failure were lower compared to the NO-DODA group, despite the full activity of the DTG and DRV/b combination being present in only 15% of the cases (Table 1).

## 4. Discussion

In this cohort of 4DR-PWH, the DTG + DRV/b combination—either as dual therapy or with at least one additional drug—was prescribed in nearly 40% of all regimens, making it one of the most frequently used strategies. The widespread use of this regimen can likely be attributed to its known potency, high genetic barrier, and the possibility of utilizing increased dosages (e.g., DRV 600 mg twice daily or DTG 50 mg twice daily) to take advantage of residual activity, despite resistance [21,22,23].

In our experience, the DODA regimen was associated with a significantly lower risk of VF, with a 77% reduction in the odds compared to regimens not containing the DTG + DRV/b combination (NO-DODA). This benefit was observed even though full activity of both DTG and DRV/b was present in only 30% of the cases within DODA. Although DODA regimens were more frequently prescribed to individuals with a more favorable genotypic profile—reflected in higher predicted activity of DRV and DTG—and NO DODA regimens were often used in more advanced patients with limited treatment options, the association between DODA and reduced risk of VF remained statistically significant after adjusting for the number of fully active drugs in the regimen and accounting for within-patient variability. Furthermore, once initiated, DODA was maintained longer than other regimens, likely reflecting its high clinical effectiveness and the improved tolerability of second-generation INSTIs and PIs. These findings confirm the efficacy of the high-genetic-barrier dual regimen in this group of 4DR-PWH, in line with a previous case series of 10 PWH from the same cohort [24].

The virological protective effect of the DTG + DRV/b combination appears to extend beyond dual therapy. In the DODA + Other group—despite a less favorable baseline genotypic resistance profile compared to the DODA group—the odds of VF, although higher than in DODA, were still 36% lower than those observed in regimens not containing the DTG + DRV/b combination (NO-DODA). Of note, full activity of both DTG and DRV/b was documented in only 15% of DODA + Other. 

One important consideration is the estimation of DTG susceptibility across groups. In fact, it is possible that DTG susceptibility was overestimated in certain cases, given the predefined criteria used when genotypic resistance testing for integrase inhibitors was unavailable—specifically, full activity (GSS = 1) was assumed for DTG and BIC even in the presence of prior failure to RAL and/or EVG. As a result, some individuals with potential underlying DTG resistance may have been treated with the DTG and DRV/b combination. In some studies, approximately 60–65% of viruses failing on raltegravir remain susceptible to dolutegravir [25,26]. In our analysis, however, we assigned full susceptibility to DTG even in individuals with prior failure on first-generation agents, likely leading to an overestimation of actual susceptibility. Therefore, the performance of DTG + DRV/b could be even better in a truly fully susceptible population. The consistently strong outcome of the DRV/b and DTG combination in the DODA + Other group remains challenging to fully explain. Although the individual activity of DTG and DRV was comparable between DODA + Other and NO-DODA, VF was notably more frequent in the latter. This observation may reflect the residual antiviral activity of DTG and/or DRV/b, which, despite the presence of resistance-associated mutations, may retain partial efficacy. 

These findings may suggest—although our study did not directly analyze resistance-associated mutations—that some patterns of partial resistance might not fully translate into treatment failure when high-genetic-barrier agents are used in optimized combinations. Moreover, it is possible that, especially in treatment-experienced individuals, the overall performance of a regimen may depend not only on the additive effects of each individual drug, but also on potential synergistic interactions within the combination—interactions that standard resistance scoring systems may not fully capture. Furthermore, unmeasured biases, potential drug interactions with other medications, and individual adherence issues may also play a role. However, it appears that DRV/b and DTG consistently perform better when used together, potentially owing to their residual activity in the presence of GRT resistance.

Regarding other factors, older age and longer ART duration were associated with reduced odds of VF, which may reflect factors such as greater treatment experience, improved self-management skills, and possibly higher adherence typically observed in older individuals, as reported in some cohorts [27,28].

As mentioned earlier, this study has several limitations, primarily due to its retrospective nature, which may have introduced a selection bias in the choice of regimen. Additionally, numerous confounding factors, such as adherence to ART, were not analyzed, and the sample size was relatively small. Moreover, we did not explore the impact of increased dolutegravir dosing (e.g., BID) or variations in DRV/b administration, which might have contributed to overcoming residual cross-resistance to first-generation INSTIs. Such analyses would have required subgroup stratification based on drug dosing, leading to excessive data granularity and a consequent loss of statistical power. Another important limitation stems from the analytical approach itself. Since the unit of analysis was the treatment regimen rather than the individual, comparisons between treatment strategies may be influenced by the fact that the same individual could contribute to multiple regimens over time. While this approach allowed us to more accurately reflect the real-life therapeutic complexity of this heavily treatment-experienced population, it also entails that regimens are not fully independent observations. Although we adjusted for key variables such as ART duration and CD4+ nadir, and accounted for individual predisposition to VF using a random intercept, residual confounding cannot be ruled out. Additionally, the absence of integrase resistance testing in some participants may have led to an overestimation of DTG susceptibility, potentially affecting the interpretation of regimen effectiveness. Although discussed above, this should be considered a methodological limitation. Finally, the use of a stringent VF definition (>50 copies/mL), although aligned with both European and national guidelines, as well as with real-life studies conducted in Italian and broader European contexts, may have led to the classification of transient low-level viremia (‘blips’) as virological failures. This approach, however, allows for better comparability with the existing literature and clinical practice in our setting.

Despite these limitations, the PRESTIGIO cohort represents the largest dataset of PWH with resistance to the four main ARV classes. To the best of our knowledge, these findings provide valuable real-world data on the efficacy of these regimens in such a challenging population.

## 5. Conclusions

In conclusion, among 4DR-PWH, the combination of DRV/b and DTG was associated with a lower probability of virological failure, even after adjusting for the number of fully active drugs. This combination remains highly effective compared to when it is not used, even when not fully supported by the activity of both drugs, or when combined with other antiretrovirals. The use of this combination in this setting—particularly the component involving protease inhibitors—is likely to decline in the future. New therapeutic options are emerging, including broadly neutralizing antibodies (bNAbs) and third-generation integrase inhibitors. These, together with the increasing availability and clinical use of recently approved drugs such as fostemsavir and lenacapavir, are collectively reshaping the treatment landscape. Nonetheless, the high efficacy of DTG and DRV/b combination remains highly relevant, particularly for fragile patients with multi-drug-resistant HIV, and should remain an important therapeutic consideration.

## Figures and Tables

**Table 1:** Number, duration, and activity of antiretrovirals included in the ART regimens analyzed in a cohort of people with 4-class drug-resistant HIV.

	DODA (n = 72)	DODA + Other (n = 264)	NO-DODA (n = 508)
Duration of the regimen in years; Median (IQR)	4.2 (1.7–5.7)	1.8 (0.6–3.5)	1.3 (0.5–2.8)
Cumulative duration of the regimen in years	280.1	656.9	1010.1
Number of fully active drugs included at each regimen initiation ^§^:			
0	14 (19.4%)	71 (26.9%)	201 (39.6%)
1	36 (50.0%)	95 (36.0%)	200 (39.4%)
2	22 (30.6%)	69 (26.1%)	87 (17.1%)
>2	0 (0.0%)	29 (11.0%)	20 (3.9%)
Percentage of regimens with full activity of DRV/b at the time of its inclusion in the regimen	34 (47.2%)	70 (26.5%)	49/207 * (23.7%)
Percentage of regimen with full activity of DTG at the time of its inclusion in the regimen	46 (63.9%)	122 (46.2%)	89/179 ** (49.7%)
Full activity of both DRV and DTG at the time of its inclusion in the regimen	22 (30.6%)	41 (15.5%)	NA

* regimens with DRV/b in NO-DODA N = 207/508 (40.7%). ** regimens with DTG in NO-DODA N = 179/508 (35.2%). ^§^ *p*-value refers to the comparison of the number of fully active drugs at regimen initiation.: DODA vs. NO-DODA (*p* = 0.001). DODA vs. DODA + Other (*p* = 0.628). DRV/b: Boosted Darunavir; DTG: Dolutegravir; DODA: regimens including DRV/b + DTG only; DODA + Other: regimens including DTG and DRV/b plus ≥1 additional antiretroviral drug. NO-DODA: regimens not including the combination of DTG and DRV/b. NA = not applicable

## Data Availability

The data presented in this study are available on request from the corresponding author.

## Abbreviations

The following abbreviations are used in this manuscript:

4DR	4-class Drug Resistance
4DR-PWH	People with HIV with 4-class Drug Resistance
ART	Antiretroviral Therapy
ARV	Antiretroviral
BIC	Bictegravir
CD4+	CD4-positive T lymphocytes
CI	Confidence Interval
DDI	Drug–Drug Interaction
DODA	Dual therapy with Dolutegravir plus boosted Darunavir
DODA + Other	Dolutegravir plus boosted Darunavir with ≥1 additional ARV
DRV/b	Boosted Darunavir (Darunavir co-administered with ritonavir or cobicistat)
DTG	Dolutegravir
EVG	Elvitegravir
GRT	Genotypic Resistance Testing
GSS	Genotypic Susceptibility Score
HTE	Heavily Treatment-Experienced
INSTI	Integrase Strand Transfer Inhibitor
IQR	Interquartile Range
NO-DODA	Regimens excluding the DTG + DRV/b combination
PWH	People with HIV
RAL	Raltegravir
VF	Virological Failure
